# Supplementary material for: Integrating Genetic and Chromosome Maps of Allium cepa: From Markers Visualization to Genome Assembly Verification
Source: Int J Mol Sci. 2022 Sep 10;23(18):10486. doi: 10.3390/ijms231810486 (PMC9504663; doi:10.3390/ijms231810486)
Supplement: Supplementary file 1 [file ijms-23-10486-s001.zip › Supplementary_Figures_S1-S11.pdf]

Supplementary figures S1-S11

Chromosome 2

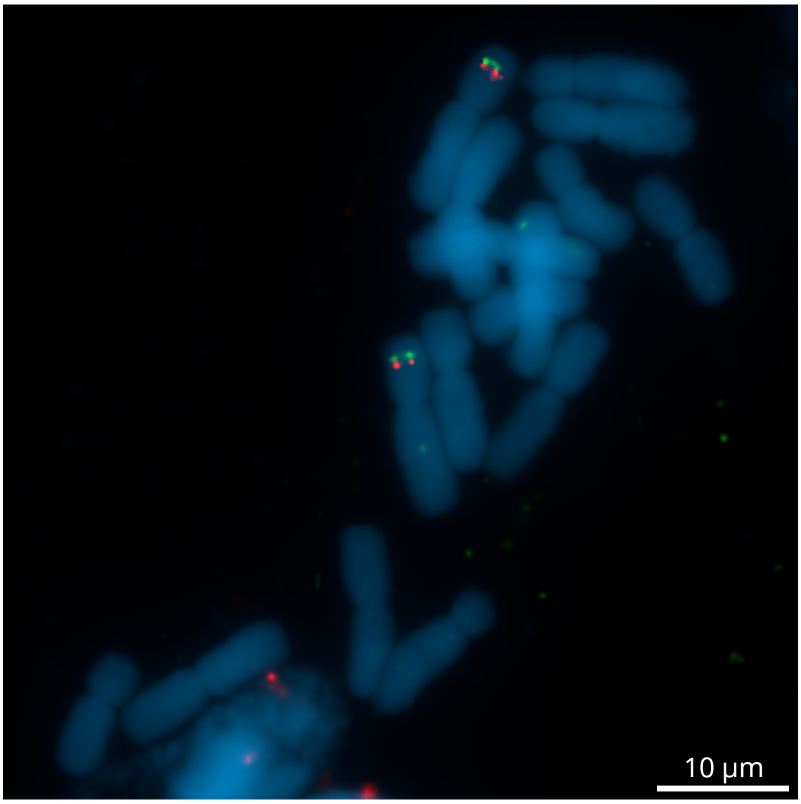

Figure S1. Dual-color Tyr-FISH on mitotic metaphase chromosome 2 of *Allium cepa* probing with Unigene572 (red) and CL5148.Contig1 (green). Scale bar - 10 μm.

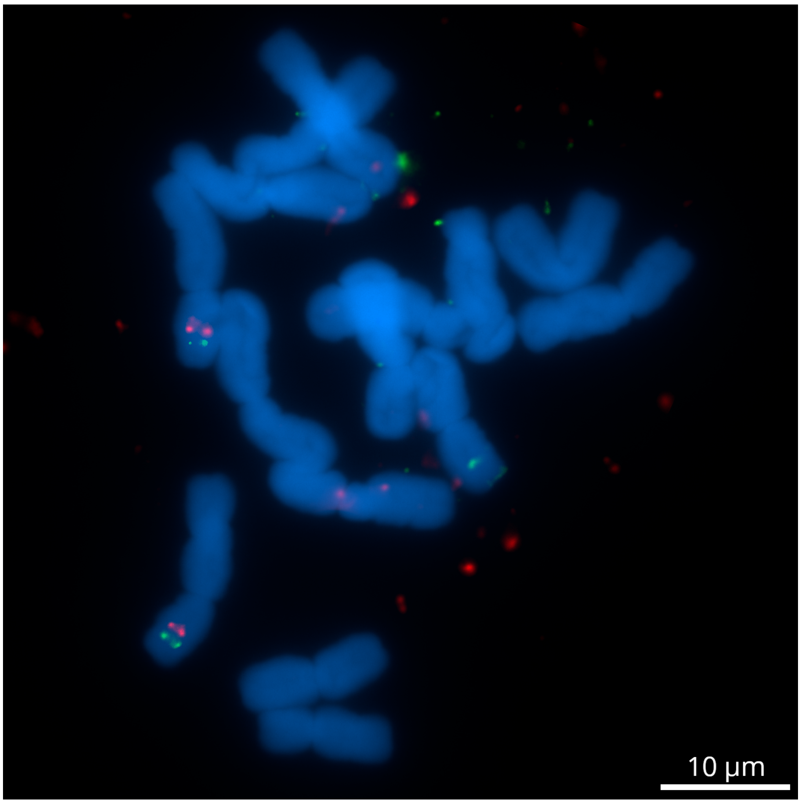

Figure S2. Dual-color Tyr-FISH on mitotic metaphase chromosome 2 of *Allium cepa* probing with CL4449.Contig1 (red) and Unigene10061 (green). Scale bar - 10 μm.

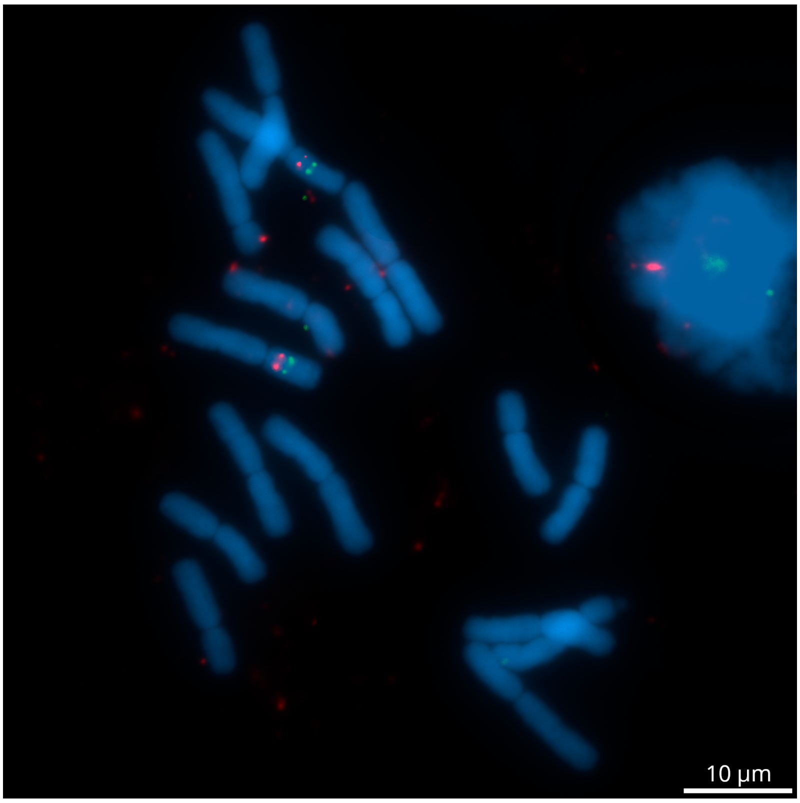

Figure S3. Dual-color Tyr-FISH on mitotic metaphase chromosome 2 of *Allium cepa* probing with Unigene27326 (red) and Unigene25645 (green). Scale bar - 10 μm.

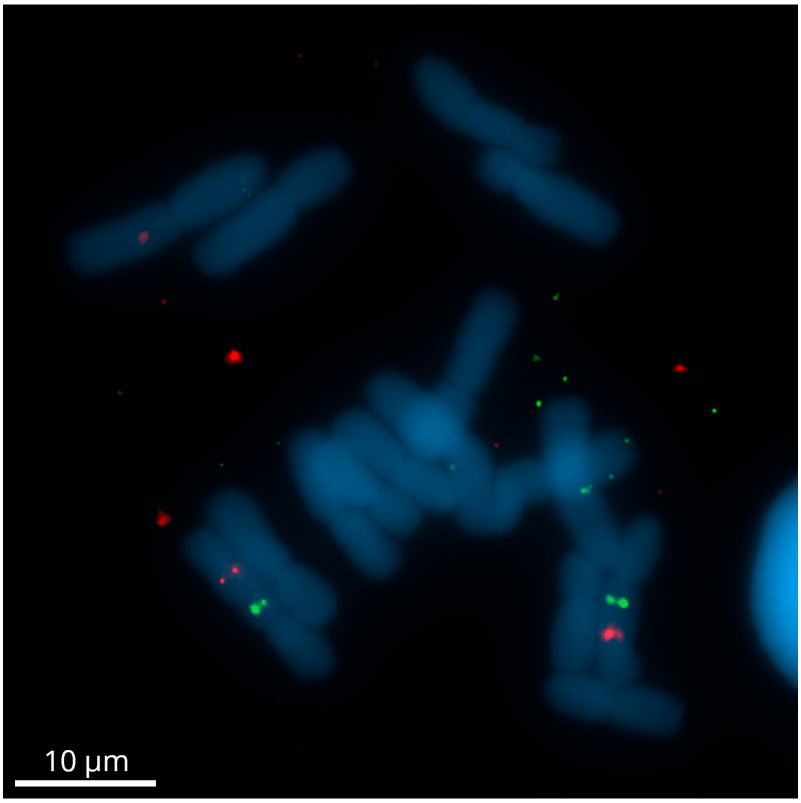

Figure S4. Dual-color Tyr-FISH on mitotic metaphase chromosome 2 of *Allium cepa* probing with Unigene5305 (red) and Unigene28713 (green). Scale bar - 10 μm.

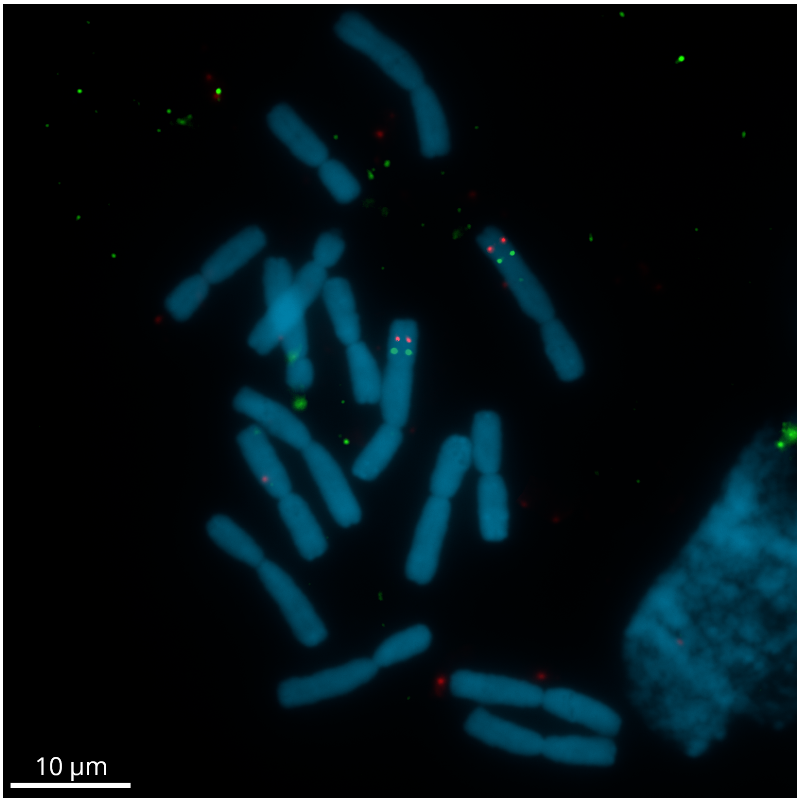

Figure S5. Dual-color Tyr-FISH on mitotic metaphase chromosome 2 of *Allium cepa* probing with Unigene23418 (red) and Unigene10683 (green). Scale bar - 10 μm.

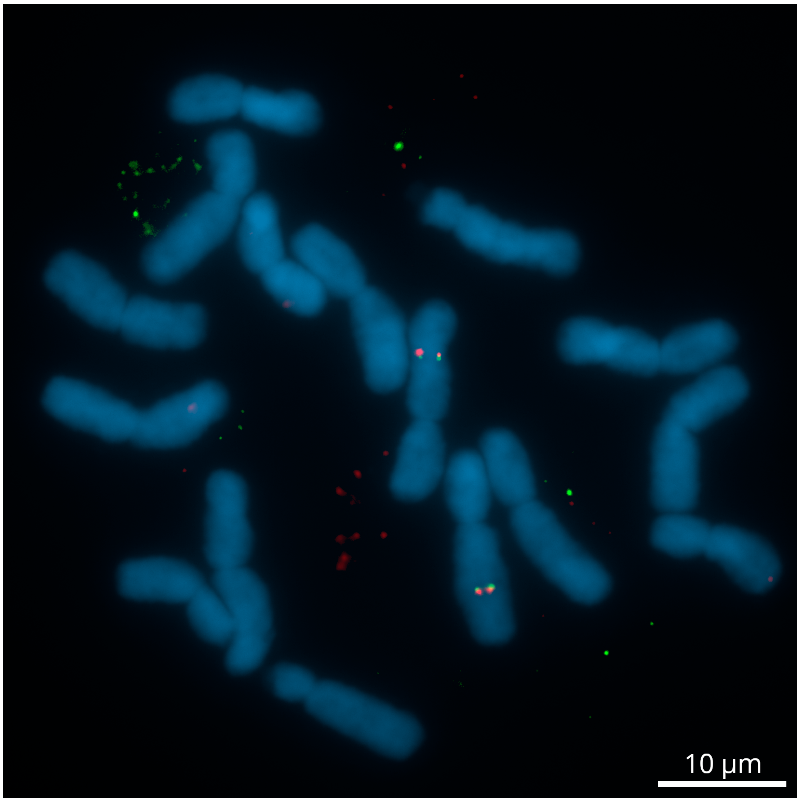

Figure S6. Dual-color Tyr-FISH on mitotic metaphase chromosome 2 of *Allium cepa* probing with *mlh1* gene (red) and Unigene5305 (green). Scale bar - 10 μm.

Chromosome 6

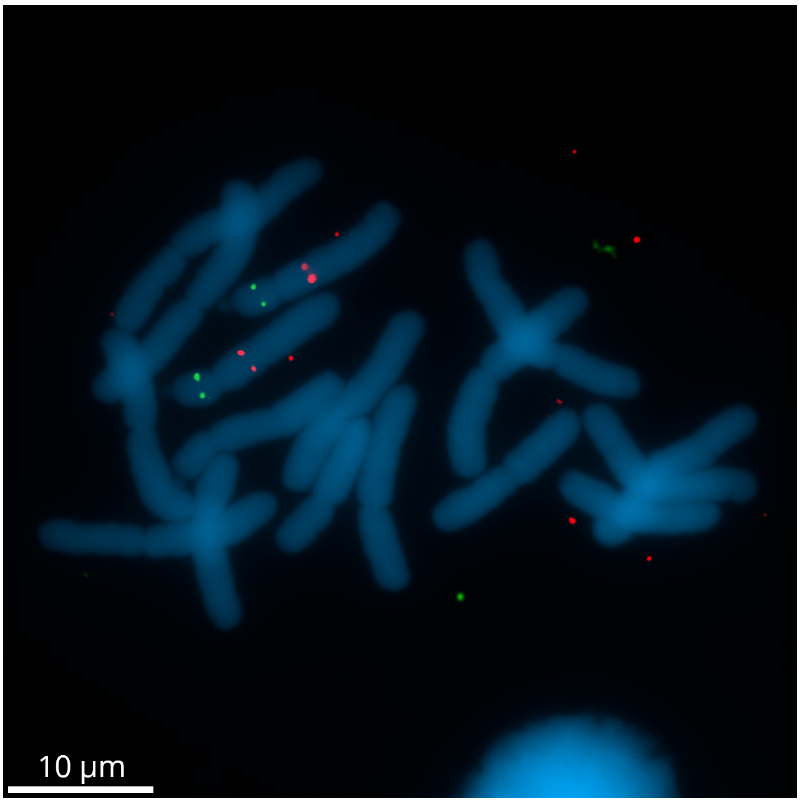

Figure S7. Dual-color Tyr-FISH on mitotic metaphase chromosome 6 of *Allium cepa* probing with Unigene13863 (red) and Unigene7941 (green). Scale bar - 10 μm.

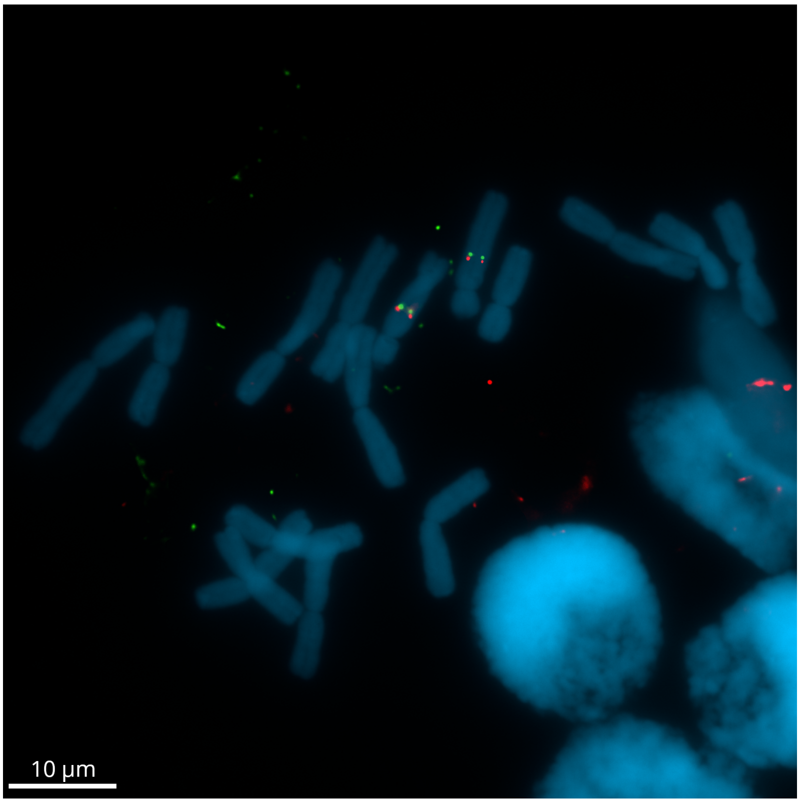

Figure S8. Dual-color Tyr-FISH on mitotic metaphase chromosome 6 of *Allium cepa* probing with Unigene49 (red) and CL4877.Contig2 (green). Scale bar - 10 μm.

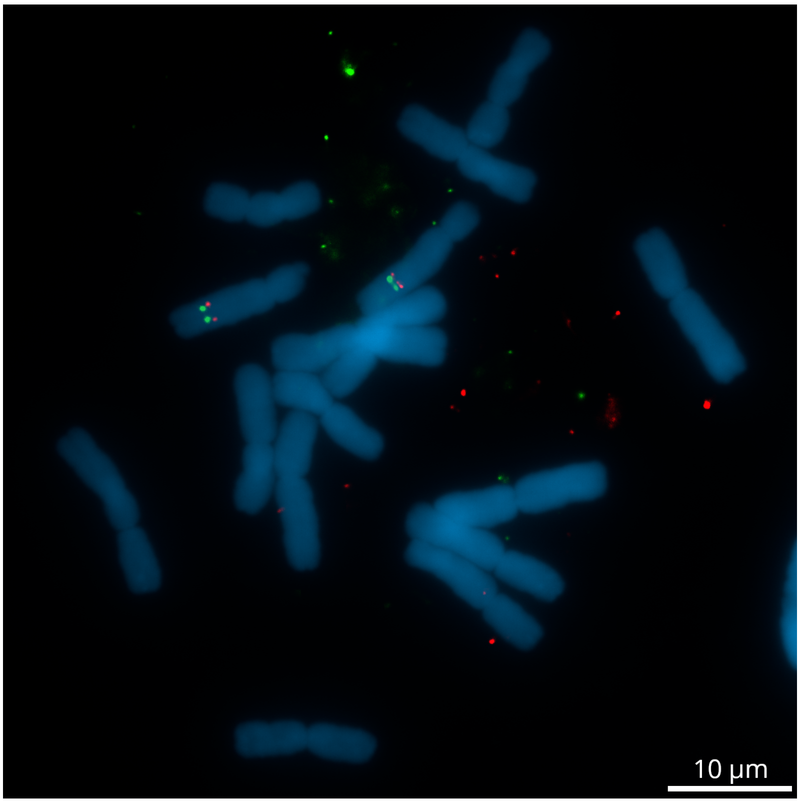

Figure S9. Dual-color Tyr-FISH on mitotic metaphase chromosome 6 of *Allium cepa* probing with Unigene22659 (red) and Unigene10558 (green). Scale bar - 10 μm.

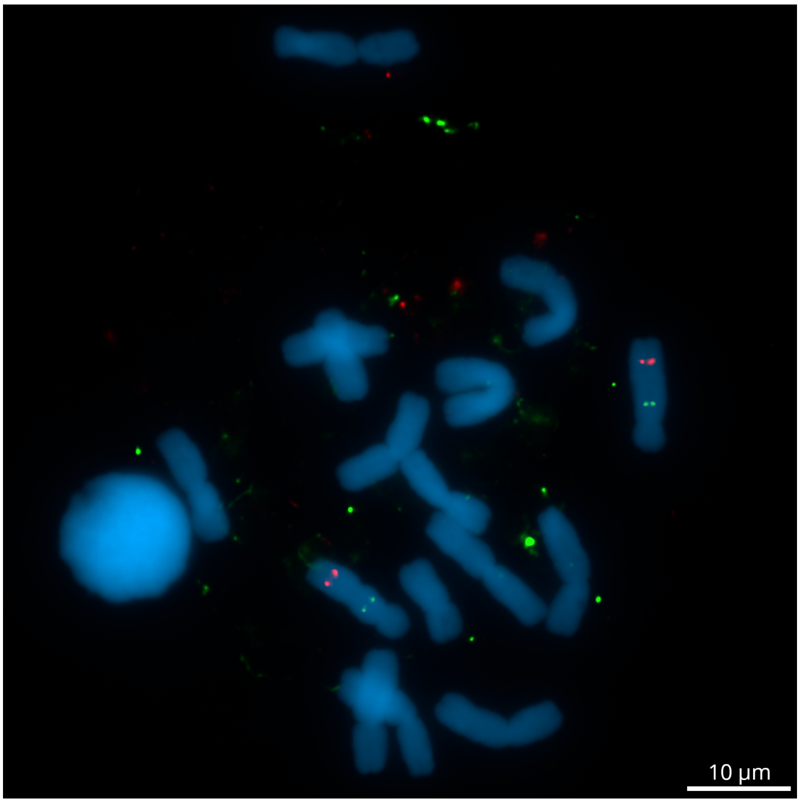

Figure S10. Dual-color Tyr-FISH on mitotic metaphase chromosome 6 of *Allium cepa* probing with Unigene28149 (red) and CL39.Contig3 (green). Scale bar - 10 μm.

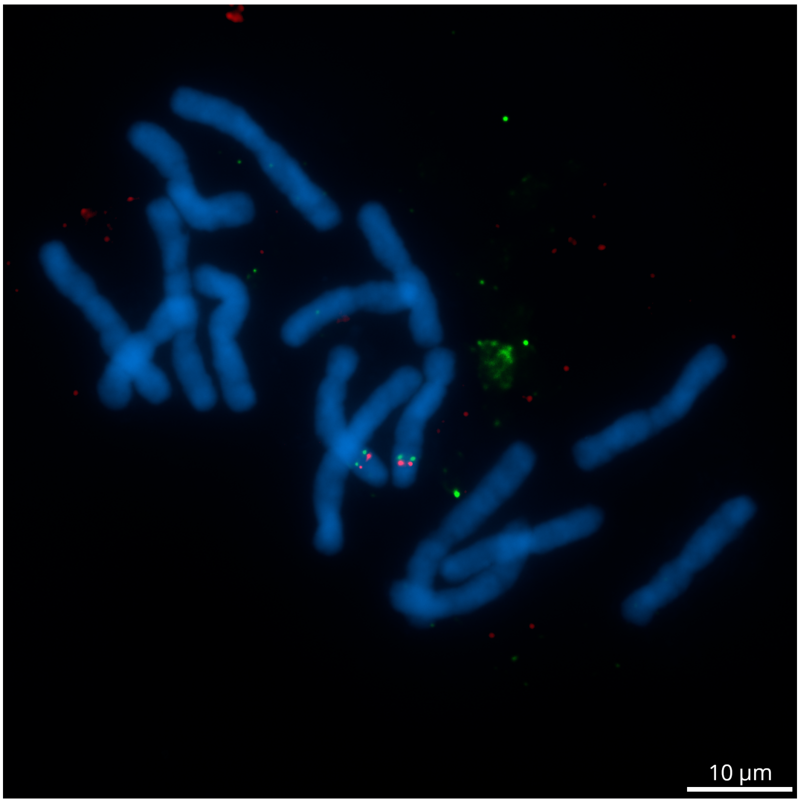

Figure S11. Dual-color Tyr-FISH on mitotic metaphase chromosome 6 of *Allium cepa* probing with Unigene8201 (red) and Unigene28149 (green). Scale bar - 10 μm.
